# Antimicrobial nitric oxide releasing gelatin nanoparticles to combat drug resistant bacterial and fungal infections†

**DOI:** 10.1039/d4na01042f

**Published:** 2025-04-08

**Authors:** Erin Myles, Raechelle. A. D'Sa, Jenny Aveyard

**Affiliations:** a School of Engineering, University of Liverpool The Quadrangle, Brownlow Hill L69 3GH UK zippy78@liverpool.ac.uk

## Abstract

Antimicrobial resistance (AMR) represents a significant global health challenge, contributing to increased mortality rates and substantial economic burdens. The development of new antimicrobial agents with dual antimicrobial and antibiofilm capabilities is crucial to mitigate AMR. Nitric oxide (NO) is a broad-spectrum antimicrobial agent which has shown promise in treating infections due to its multiple antimicrobial mechanisms. However, the high reactivity of NO poses a challenge for effective delivery to infection sites. We investigated the antimicrobial and antibiofilm capabilities, and the shelf life, of NO-releasing gelatin nanoparticles (GNP/NO) against three common hospital-acquired pathogens: *Staphylococcus aureus*, *Escherichia coli*, and *Candida albicans*. The synthesised GNP/NO were found to be cytocompatible and exhibited significant antimicrobial and antibiofilm efficacies against the tested pathogens in both nutrient-rich and nutrient-poor conditions. Furthermore, we found that the antimicrobial capabilities of GNP/NO were maintained for up to 6 months post synthesis, against *Staphylococcus aureus* (2.4 log), *Escherichia coli* (1.2 log) and *Candida albicans* (3 log) under nutrient-poor conditions. Our study demonstrates the use of a novel broad-spectrum antimicrobial with a prolonged shelf life for the treatment of infections. These findings offer an effective alternative to traditional antibiotics which would contribute to mitigating the current global AMR threat resulting from antibiotic overuse.

## Abstract 2

This proof-of-concept study outlines the synthesis/characterisation of homogenous nitric oxide releasing nanoparticles, demonstrating antimicrobial and antibiofilm capabilities against *Staphylococcus aureus*, *Escherichia coli* and *Candida albicans*.

## Graphical abstract

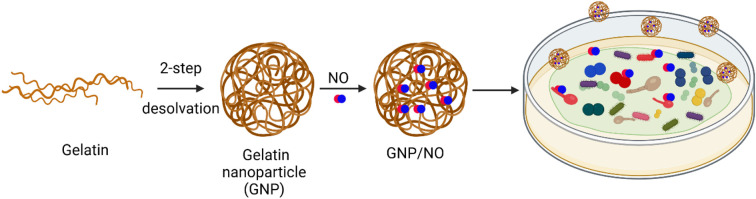

## 1. Introduction

Antimicrobial resistance (AMR) represents a significant global health challenge, contributing to approximately 700 000 deaths annually due to hospital-acquired drug-resistant infections.^1^ The rise in antibiotic-resistant bacteria places a substantial economic burden on healthcare systems, with costs reaching an estimated $1.5 billion in Europe.^2^ Furthermore, the O'Neill report^3^ concluded that, without decisive action, AMR could result in 10 million deaths a year, with a global economic cost of $100 trillion by the year 2050. Given the rising global threat of AMR and its impact on healthcare systems, it is vital that new antimicrobial agents with both antimicrobial and antibiofilm capabilities are developed to mitigate the effects of AMR.

Nitric oxide (NO) is a broad-spectrum antimicrobial agent which is endogenously produced by macrophages within the immune system, in response to pathogens. The antimicrobial capabilities of NO are attributed to its ability to react readily with oxygen, superoxide (O_2_^−^) and hydrogen peroxide (H_2_O_2_) to form highly reactive nitrogen and oxygen species (RNS and ROS), such as peroxynitrite, nitrogen dioxide and dinitrogen tetroxide. The presence of RNS and ROS over time leads to intracellular nitrosative and oxidative stress within bacterial cells, causing DNA alterations, lipid peroxidation and enzyme inactivation, making NO an effective antimicrobial agent.^4,5^ Owing to the multiple antimicrobial mechanisms by which NO can inactivate microorganisms, there has been intense interest in the use of NO-releasing delivery systems as a potential therapy for treating infections.^6,7^ Moreover, studies by Privett^8^ and Grayton^9^ have demonstrated that exposure to sublethal dosages of exogenous NO is unlikely to induce resistance in bacteria or fungi, likely due to the multifaceted antimicrobial mechanisms of NO.^5,8,9^

Despite its antimicrobial properties, the radical nature of NO makes it highly reactive, resulting in a short shelf life (<10 s). Therefore, targeted NO delivery to the site of infection is challenging.^10^ To address this issue NO donors, such as *N*-diazeniumdiolates have been developed to improve the storage capabilities of NO and enhance delivery of therapeutic doses to target sites. *N*-diazeniumdiolate compounds, characterised by their diolate [N–(O)N=O] functional group, are formed by the reaction of amines with NO under high pressure in the absence of oxygen. This reaction forms a diolate group bound to a nucleophile adduct to form on a nitrogen atom within the amine.^11^ The rate of NO release from *N*-diazeniumdiolates is highly dependent on the p*K*a of the amine group to which they are attached. Primary amines typically produce less stable *N*-diazeniumdiolates with rapid release rates.^12^ Consequently, secondary amines and polyamines are more commonly used in biomaterials as they enhance the shelf life and improve the release kinetics of *N*-diazeniumdiolates.^13,14^

Gelatin, derived from the hydrolysis of collagen, and is a widely used biomacromolecule within both the food and pharmaceutical industries, due to its biocompatibility and cost-effectiveness.^15^ In clinical settings, gelatin is commonly used as a vaccine stabiliser, such as in the inhaled influenza vaccine to improve the stability and shelf-life of the product.^16^ As gelatin is derived from collagen its structure comprises of multiple amino acid groups, this allows for many chemical modifications and covalent attachment, making it an ideal candidate for use as a drug delivery vehicle.^17^ The use of gelatin-derived biomaterials has been well documented for a range of applications, including tissue engineering, cancer treatments and wound healing.^18–20^ Furthermore, Li *et al.*^21^ has shown that electrospun polycaprolactone (PCL)/gelatin blended wound dressings (PCL : G. 25 : 75 wt%) can be functionalised with *N*-diazeniumdiolates, as the polyamine composition of gelatin provides multiple tethering sites for the NO donor. The electrospun wound dressing significantly reduced the presence of both *Pseudomonas aeruginosa* and *Staphylococcus aureus*, reducing the risk of infections.

Gelatin nanoparticles (GNPs) have been extensively studied as a drug delivery system for a range of applications, including cancer treatment,^22^ tissue engineering,^23^ and vaccine delivery.^24^ However the use of GNP for the delivery of NO has not been investigated, therefore this study assessed the antimicrobial efficacy of *N*-diazeniumdiolate-releasing gelatin nanoparticles (GNP/NO).

We investigated the antimicrobial and antibiofilm capabilities of GNP/NO against three common hospital-acquired pathogens. This proof-of-concept study outlines the synthesis of homogenous GNP/NO, which demonstrated antimicrobial and antibiofilm capabilities against, *Staphylococcus aureus*, *Escherichia coli* and *Candida albicans* within a cytocompatible range, in both nutrient-rich and nutrient-poor conditions. Notably, the nanoparticles maintained their antimicrobial efficacy after 6 months of storage. These results emphasise the potential of GNP/NO as an effective infection treatment, which may help to mitigate the current global AMR threat caused by antibiotic overuse.

## 2. Experimental section

### 2.1 Gelatin nanoparticle synthesis

Gelatin nanoparticles (GNPs) were synthesised using a modified two-step desolvation method developed by Coester.^25^ Briefly, 1 g gelatin type A (300 bloom) from porcine skin (Sigma-Aldrich) was dissolved in 20 mL dH_2_O and heated from ambient temperature to 60 °C while stirring (1000 rpm). After reaching 60 °C, 20 mL of acetone (Thermo Scientific) was added to the solution, forming two distinct layers of high- and low-molecular weight components. The top layer (containing low molecular weight components) was discarded, after which 20 mL dH_2_O was added to the remaining layer and stirring was resumed and maintained at 60 °C. The pH was adjusted to pH 3.5 with the addition of HCl and NaOH (Sigma-Aldrich). The temperature was lowered to 40 °C, and stirring was reduced (500 rpm), after which 70 mL of acetone was added dropwise over 14 min. To crosslink the particles, 80 μL of 50% glutaraldehyde (Sigma-Aldrich) was added and stirred for 1 h at room temperature. The solution was statically incubated for 12 h at ambient temperature.

**Fig. 1:**
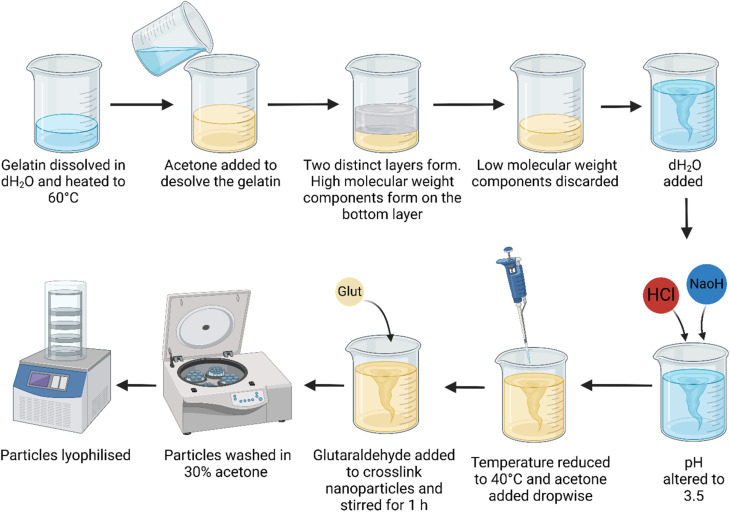
Schematic of the two-step desolvation method used to synthesise gelatin nanoparticles. Created with BioRender.com.

The particles were centrifuged at 10 500 rpm (Thermo-Scientific Heraeus Megafuge 16R) for 10 min and washed in 30% acetone, this step was then repeated a further 3 times. After the final wash, GNPs were resuspended in dH_2_O and frozen before lyophilisation (ScanVac Cool Safe, La60 Gene). The particles were then stored in a refrigerator at 5 °C until required. A schematic representation of this method is depicted in Fig. 1.

### 2.2 Gelatin nanoparticle size and zeta potential

The particle size and zeta potential of GNPs were measured using a Zetasizer Nano ZS, (Malvern Analytical Instruments). The particle size was measured before lyophilization at 25 °C with a scattering angle of 173°. The volume % per batch was also recorded, allowing the assessment of interbatch viability. Each sample was measured in triplicate and the hydrodynamic diameter (*Z*-average) is presented as mean ± standard deviation. The polydispersity index (PDI) is a measure of the size distribution of the nanoparticle population.

To measure the zeta potential of the particles, 2 mg of lyophilised GNPs were suspended in 1 mL dH_2_O, placed into a capillary cuvette (Malvern Panalytical), and measured using a Zetasizer Nano ZS.

### 2.3 Scanning electron microscopy

The morphology and diameter of GNPs after lyophilisation were determined by SEM. Synthesised GNPs were placed onto carbon tape and sputter-coated (Q150T ES Sputter Coater, Quorum, East Sussex, UK) with gold to increase the surface electrical conductivity. GNP were imaged using FSEM (JSM 7001 F FEGSEM; JEOL, Tokyo, Japan) with an accelerating voltage of 10 kV and a working distance of 9 mm.

All images were processed using ImageJ software, and an average particle size was obtained from each image (*n* = 30).

### 2.4 Gelatin nanoparticle functionalisation with *N*-diazeniumdiolate

**Fig. 2:**
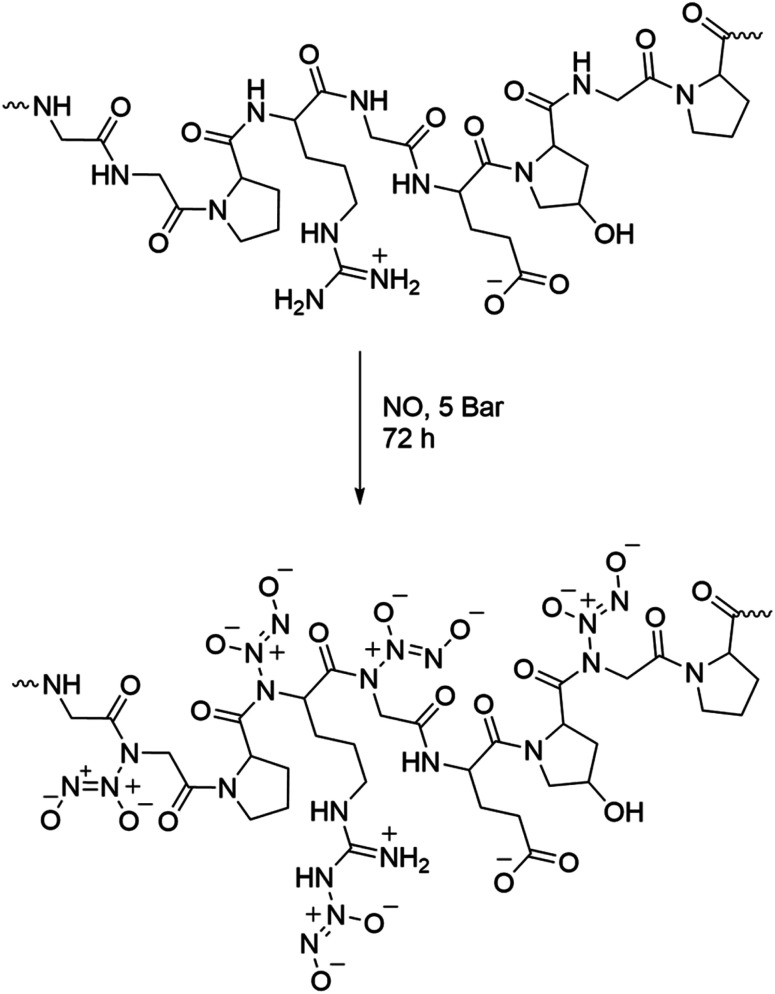
Structure of gelatin and functionalised gelatin. Top image depicts the structure of gelatin, and the bottom image shows the structure of gelatin with *N*-diazeniumdiolate tethered, following functionalisation with NO for 72 h at 5 bar.

The functionalization of GNPs with *N*-diazeniumdiolates in an NO reactor, as previously reported.^26,27^ Briefly, lyophilised GNPs were placed in a stainless-steel Parr bomb, which was purged six times with argon at 5 bar to remove any residual oxygen. The Parr bomb was subsequently filled with NO at 5 bar for 72 h to functionalise the particles with *N*-diazeniumdiolates (Fig. 2). Subsequently, six argon purges were conducted to remove residual NO from the Parr bomb. The diazeniumdiolate-functionalised GNPs (GNP/NO) were stored in a box desiccator in a freezer (−20 °C) until required. In this reaction *N*-diazeniumdiolate are tethered onto primary and secondary amine in the gelatin structure as shown in Fig. 2.

### 2.5 pH of media with addition of GNP/NO7

The effects of GNP/NO on the pH of different media were assessed by placing a pH probe (Jenway Benchtop 3510) in 5 mL PBS, DMEM, LB or TSB at ambient temperature. After which GNP/NO7 was added to each medium and the initial pH change was recorded. The pH of each medium containing GNP/NO7 was recorded after at 1, 2, 4, 18 and 24 h.

### 2.6 Chemiluminescence of GNP/NO in media

The NO release from GNP/NO under different conditions was measured using a chemiluminescence NO Analyser (Sievers, 280i). A weight of 2.5, 5 or 7 mg of GNP/NO (referred to as GNP/NO2.5, GNP/NO5 and GNP/NO7) were placed into a three-neck flask containing 5 mL phosphate buffered saline (PBS; Sigma-Aldrich), Luria broth (LB: Oxoid) Tryptic Soya broth (TSB; BD Difco) or Dulbecco's Modified Eagle's Medium (DMEM; Gibco), depending on the condition being assessed. The NO release of GNP/NO in DMEM was undertaken without the presence of foetal bovine serum (FBS), as the proteins causes the media to froth in the sample vessel, impacting on the accuracy of the readings. Nitrogen gas was then continuously bubbled into the vessel at a flow rate of 200 mL min^−1^ to sweep the NO released by the sample from the headspace and into the detection cell. The NO release was measured over 24 h, at ambient temperature.

Measurements were conducted at 0 (*T* = 0), 3 (*T* = 3) and 6 months (*T* = 6) time points to assess the stability of NO release from GNP/NO, between time points GNP/NO were stored in a freezer at −20 °C. Measurements at *T* = 0 and *T* = 3 were conducted in triplicate (*N* = 3), whilst those measurements at *t* = 6 were performed only once (*N* = 1).

### 2.7 Stability of the antimicrobial efficacy of GNP/NO

Following functionalisation, the antimicrobial efficacy of GNP/NO was assessed against *Staphylococcus aureus* NCTC 12981 (PHE), *Escherichia coli* NCTC 9001 (PHE) and *Candida albicans* NCYC 1363 (PHE) in both PBS (nutrient-poor) and LB or TSB (nutrient-rich). The antimicrobial efficacy of three GNP/NO concentrations (2.5, 5, and 7 mg) were assessed in this paper over a period of 6 months. Three concentrations were studied to ascertain the minimum dose required to elicit an antimicrobial response, whilst maintaining cytocompatibility and stability during storage.

Microbial cultures were grown in 15 mL LB and incubated at 37 °C with agitation (150 rpm) for 18 h, except for *C. albicans* which was grown in 15 mL TSB. The cultures were centrifuged at 3500 rpm for 10 min, and the supernatant was discarded. The pellet was resuspended in sterile PBS, to achieve an OD^600^ = 1 (Hitachi, U-2900, Tokyo, Japan). The bacterial suspension was diluted 1 : 100 in PBS for nutrient-poor conditions, and in LB or TSB for nutrient-rich condition to obtain a final cell concentration of ∼10^6^ CFU mL^−1^. Following this, 1 mL cell suspension was placed into microcentrifuge tubes containing either 2.5, 5 or 7 mg GNP/NO, referred to as GNP/NO2.5, GNP/NO5 and GNP/NO7. Non-functionalised GNP of the same weight were used as controls (GNP2.5, GNP5 and GNP7), and an additional positive control containing bacterial suspension only was used and incubated at 37 °C with agitation (150 rpm).

Antimicrobial efficacy was evaluated at 4 h to assess the immediate antimicrobial effects of GNP/NO and at 24 h to examine its sustained effects on bacterial and fungal populations. These time points were chosen as they align with common antimicrobial susceptibility testing methods. Following incubation, 100 μL was taken from each microcentrifuge tube and serially diluted 1 : 10 six times in PBS. A 20 μL aliquot from each dilution were plated onto Luria broth agar (LBA; Sigma-Aldrich) in triplicate and incubated for 24 h at 37 °C, except for *C. albicans* which was plated onto tryptic soya agar (TSA; BD Difco). The number of visible colonies was counted to determine the total number of viable microorganisms per mL (CFU mL^−1^). All experiments were conducted in triplicate (*N* = 3).

This experiment was repeated at *T* = 0, *T* = 3 and *T* = 6 to assess the long-term stability all the GNP/NO concentrations and to fully optimise the antimicrobial efficacy of GNP/NO.

### 2.8 Stability of the antibiofilm efficacy of GNP/NO

A 10^6^ CFU mL^−1^ bacterial suspension of *E. coli* or *S. aureus* in LB was obtained, and 150 μL aliquots were placed into two 96-well MBEC biofilm inoculator (Innovotech), which were then sealed with Parafilm and incubated at 37 °C with agitation (150 rpm). Following this, the plate lid containing pegs upon which the biofilm had adhered, was removed and placed in 200 μL of sterile PBS and left to stand for 1 min, to remove and residual planktonic bacteria, then the lid was immersed in 200 μL LB broth (nutrient rich conditions) or PBS (nutrient poor conditions) containing 0.5, 1 or 1.4 mg of GNP/NO. Non-functionalised GNP of the same weights were used as controls (GNP2.5, GNP5 and GNP7), and an additional positive control containing bacterial suspension only was used and incubated at 37 °C with agitation (150 rpm) which was used as a control. The plate was again sealed with Parafilm and incubated for an additional 4 or 24 h.

The plate lid was subsequently rinsed with PBS and immersed in a new 96-well plate containing 200 μL media. The plate was sonicated (VWR, USC100 TH Ultrasonic Bath) for 10 min to remove biofilms from the pegs. A 20 μL aliquot was removed from each test well and serially diluted (1 : 10) to a factor of eight, and 20 μL of each dilution was plated onto LB agar and incubated for 24 h at 37 °C. The number of visible colonies were counted, and CFU mL^−1^ was calculated.

Antimicrobial efficacy was evaluated at *T* = 0, *T* = 3 and *T* = 6 to assess the longevity antimicrobial stability of the particles. However, these experiments were not conducted against *C. albicans* due to the inability of this strain to form biofilms.

### 2.9 Statistical analysis

All values are expressed as mean ± SD in their log_10_ form. Significant differences between samples obtained from antimicrobial testing of GNP/NO were analysed as follows; normality of samples was assessed using the Shapiro–Wilk test. One-Way ANOVA was performed followed by a post-hoc Dunnett test to determine significance. The alpha level was set at 5%, and a *p*-value ≤ 0.05 was considered significant for all tests. Statistical analyses were performed using SPSS software (IBM).

### 2.10 Cytotoxicity of GNP/NO against L929

The ISO-10993 indirect (leachate) protocol was used to assess the cytotoxicity of GNP/NO against the murine fibroblast L929 cell line (ECACC 85011425). Briefly, cells were incubated in 15 mL DMEM containing 10% foetal bovine serum (FBS; Gibco) 37 °C under 5% CO_2_ until 90% confluency was reached. The cells were trypsinised to detach them from a T-flask (Thermo Fisher Scientific) and centrifuged at 1000 rpm for 5 min. The supernatant was discarded, and the pellet was resuspended in fresh medium to remove trypsin. Cells were seeded at density of 1 × 10^5^ cells per well and were subsequently incubated for 24 h at 37 °C under 5% CO_2_. Leachates were formed by adding GNP/NO2.5, GNP/NO5 or GNP/NO7, or non-functionalised GNP2.5, GNP5 and GNP7 controls to 1 mL of DMEM in a microcentrifuge tube and incubated at 37 °C for either 4 or 24 h. After incubation, the spent medium was aspirated from the 96-well plate and replaced with 100 μL of leachates, which were incubated for a further 24 h. The leachates were then aspirated, and the cells were rinsed once with PBS to remove any residual media. A 100 μL 1 mg mL^−1^ MTT (Sigma-Aldrich) solution was added to each well and incubated for an additional 4 h to allow the formation of formazan crystals. Following this, 100 μL DMSO (Sigma-Aldrich) was added to each well and left to stand on a plate rocker (Gyro Rocker Shaker mini, Cole-Palmer) for 20 min to solubilise the crystals. A plate reader (Agilent BioTek Synergy Microplate Reader) was used to measure the colorimetric change within each well at an absorbance of 570 nm. All experiments were conducted in triplicate, and a cell viability below 70% was considered cytotoxic.

## 3. Results

### 3.1 Gelatin nanoparticle synthesis and characterisation

The 2-step desolvation method, originally developed by Coester^25^ was used to synthesise GNP. Briefly, gelatin was heated and desolved by the addition of acetone to separate the high and low molecular weight components. Following this, the pH was adjusted, and a second desolvation step was performed by the addition of acetone, resulting in the formation of GNP. The hydrodynamic diameter of GNP, measured by DLS was found to be 226.9 ± 18.8 nm (PDI 0.09 ± 0.04) and the zeta potential of GNP post lyophilisation was +10.9 ± 0.9 mV, when measured at pH 7.0. Little inter-batch variability was observed, as shown in Fig. S1.†

**Fig. 3:**
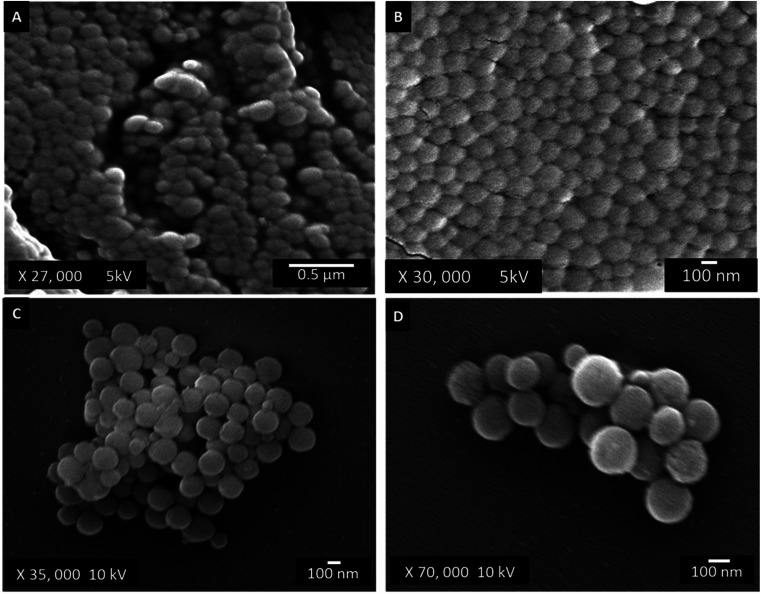
Micrographs of GNP taken at 5 kV, (A) ×27, 000 (B) ×30 000 and at 10 kV, (C) ×35 000 and (D) ×70 000. The contrast and brightness of the images have been altered to improve the visibility of the particles; all images are representative of the sample.

Scanning electron microscopy was used to characterise the morphology and size of the GNP after lyophilisation (Fig. 3a–d). The particles were homogenous and spherical in structure. The particle diameter of the lyophilised GNP, measured *via* SEM was calculated to be 190 ± 8 nm.

### 3.2 pH of biologically relevant media with GNP/NO

**Fig. 4:**
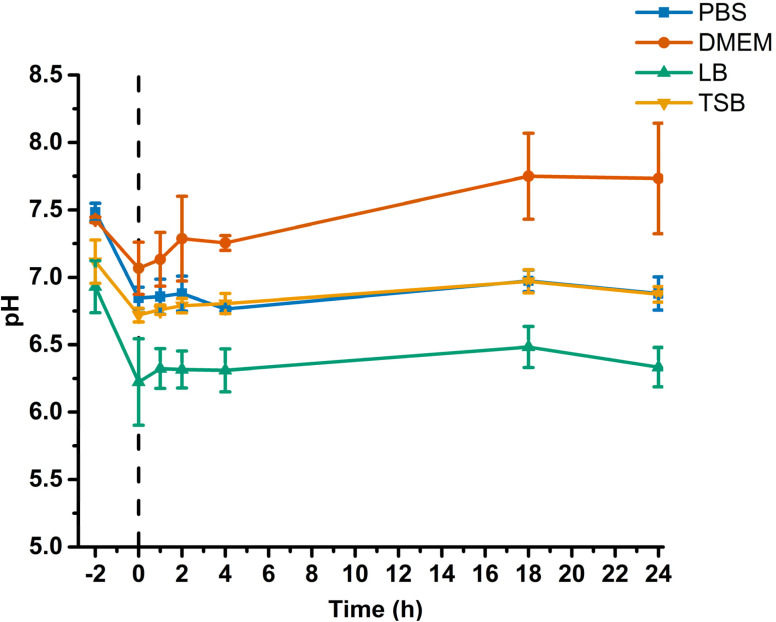
The measurements show the initial pH of the media (−2 h), after the addition of GNP/NO7 (0 h), indicated by the dashed line and the subsequent pH values at 1, 2, 4, 18 and 24 h after the addition of GNP/NO. pH was measured in PBS (blue), DMEM (red), LB (green) and TSB (yellow).

Initial work by Keefer *et al.*^28^ demonstrated that the decomposition of *N*-diazeniumdiolate is initiated by the protonation of the N–N bond, which can release up to 2 moles of NO per 1 mole of *N*-diazeniumdiolate, therefore the pH of the test medium is an important factor when considering NO release rates from *N*-diazeniumdiolates. For this reason, the pH of all media used in this study (PBS, DMEM, LB and TSB) were measured both before and after the addition of GNP/NO7 (Fig. 4), to better understand the impact of pH on the release of NO from GNP/NO, prior to the addition of GNP/NO7, it was observed that all media had a relatively neutral pH, ranging from 7.48 ± 0.07 (PBS) to 7.11 ± 0.16 (LB). After the addition of GNP/NO7, the pH of all media tested decreased, with the largest and smallest reduction exhibited by LB (pH 6.22 ± 0.32) and PBS (pH 6.85 ± 0.08). Interestingly over the 24 h measuring period, the pH of each medium increased close its initial starting pH, with the exception of DMEM which exceeded its initial starting pH (6.33 ± 0.15).

### 3.4 NO release in media

To understand the influence of media pH on NO release from GNP/NO, the NO payload was determined using a chemiluminescence nitric oxide analyser. Varying concentrations of particles (GNP/NO2.5, GNP/NO5, and GNP/NO7) were placed in PBS, LB, TSB or DMEM and the NO release was measured over 24 h.

**Fig. 5:**
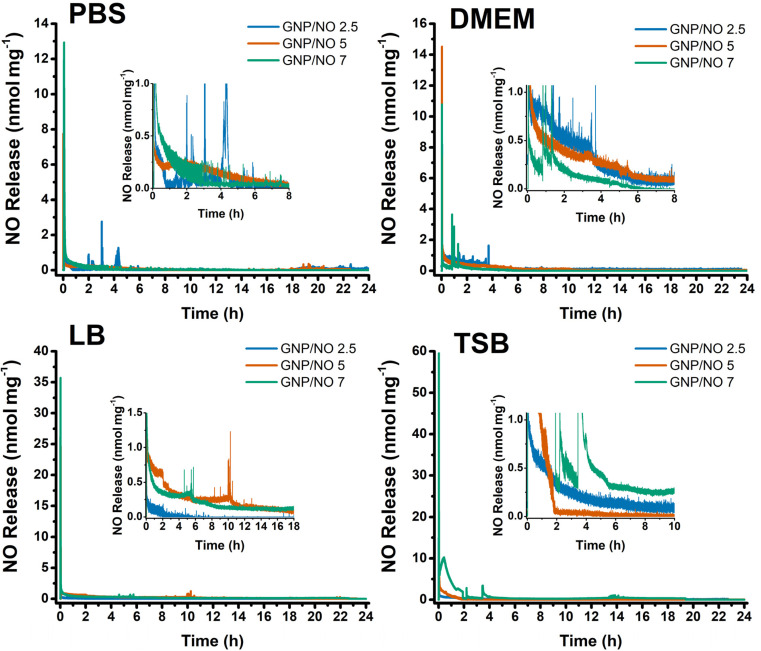
Chemiluminescence measurements of GNP/NO in PBS, DMEM, LB and TSB. The NO release profiles of GNP/NO2.5 (blue), GNP/NO5 (red) and GNP/NO7 (green). The NO release profile is representative of one run, and the experiment was performed three times (*N* = 3).

As stability is an important factor to consider when assessing the potential of new drug candidates^29^ the long-term stability and shelf life of the particles was also investigated. The NO release from GNP/NO was measured at *t* = 0 (initial measurement) and after *t* = 3 months and *t* = 6 months of storage. The release profiles of GNP/NO for each medium at *t* = 0 is shown in Fig. 5.

### 3.5 NO release in nutrient-poor conditions

The NO release kinetics for GNP/NO in nutrient-poor conditions (PBS) were measured at three timepoints (*t* = 0, *t* = 3 and *t* = 6) and are summarised in Table 1. The table includes the total NO released over the 24 h measurement period ([NO]tot), maximum NO release ([NO]max), the time taken to achieve [NO]max ([NO]tmax) and total duration of release ([NO]td).

**Table 1:** NO release kinetics of GNP/NO in PBS

Sample	Month	[NO]tot^a^ (μmol mg^−1^)	[NO]max^b^ (nmol mg^−1^)	[NO]tmax^c^ (min)	[NO]td^d^ (h)
GNP/NO2.5	0	9.21 ± 3.80	1.79 ± 0.76	0.35 ± 0.23	12.83 ± 6.66
	3	8.83 ± 0.75	10.33 ± 4.17	0.47 ± 0.19	10.21 ± 0.83
	6	0.46	4.26	0.20	0.34
GNP/NO5	0	6.09 ± 3.16	11.02 ± 4.83	0.12 ± 0.02	8.14 ± 0.68
	3	22.18 ± 4.51	12.93 ± 0.00	0.185 ± 0.01	11.35 ± 0.01
	6	2.25	8.16	0.27	3.23
GNP/NO7	0	7.65 ± 4.68	23.29 ± 29.77	0.44 ± 0.23	7.95 ± 4.54
	3	2.19 ± 0.34	10.46 ± 4.75	0.16 ± 0.10	4.96 ± 2.58
	6	0.83	4.77	0.38	1.72

a The total NO release from the sample over time.

b The highest release burst from the sample.

c The time taken for a sample to achieve [NO]max.

d The total duration of release for a sample.

*T* = 0: at *t* = 0, it was revealed that the [NO]tot was not proportional to the GNP/NO concentration as GNP/NO2.5, GNP/NO5 and GNP/NO7 released 9.21 ± 3.80, 6.09 ± 3.16 and 7.65 ± 4.68 μmol mg^−1^, respectively. As well as demonstrating the highest [NO]tot release, GNP/NO2.5 also exhibited the longest [NO]td (12.83 ± 6.66 h) in PBS.

*T* = 3: at *t* = 3 a reduction in [NO]tot concentration for GNP/NO2.5 (8.83 ± 0.75) and GNP/NO7 (2.19 ± 0.34 μmol mg^−1^) was observed, however interestingly the opposite was seen for GNP/NO5 (22.18 ± 4.51 μmol mg^−1^).

*T* = 6: the decrease in NO release from GNP/NO over time was also reflected in the results at *t* = 6, which found that the [NO]tot for all GNP/NO concentrations decreased considerably compared with those at *t* = 0.

### 3.6 NO release in nutrient-rich conditions

Hunter *et al.*^30^ demonstrated that NO release kinetics are significantly influenced by the composition of the surrounding medium, and observations from this study revealed pH variations in the biologically relevant test media, which may cause variations in the decomposition of *N*-diazeniumdiolates. We assessed the NO release kinetics of GNP/NO in LB, TSB and DMEM, with an aim to provide a greater understanding on the effects of media on NO release kinetics from GNP/NO, and consequently the antimicrobial efficacy of the nanoparticles under different conditions. To investigate the shelf-life of GNP/NO the NO release measurements were performed at *t* = 0, *t* = 3 and *t* = 6 under nutrient-rich conditions.

#### 3.6.1 NO release in LB

The NO release profiles of GNP/NO in LB at *t* = 0 are displayed in Fig. 4, and the NO release kinetics at each time point were calculated and are summarised in Table 2.

**Table 2:** Stability of NO release kinetics from GNP/NO in LB

Sample	Month	[NO]tot^a^ (μmol mg^−1^)	[NO]max^b^ (nmol mg^−1^)	[NO]tmax^c^ (min)	[NO]td^d^ (h)
GNP/NO2.5	0	14.55 ± 11.21	44.45 ± 19.91	0.16 ± 0.02	7.63 ± 4.82
	3	6.06 ± 5.63	12.80 ± 0.18	0.24 ± 0.06	7.50 ± 7.23
	6	1.64	7.38	0.27	2.73
GNP/NO5	0	19.09 ± 0.67	19.00 ± 5.66	0.21 ± 0.12	9.30 ± 1.67
	3	5.97 ± 5.25	6.66 ± 2.08	0.21 ± 0.06	7.57 ± 5.03
	6	1.18	5.95	0.14	1.94
GNP/NO7	0	19.96 ± 1.76	22.4 ± 9.75	0.09 ± 0.06	19.55 ± 2.31
	3	8.27 ± 6.42	13.11 ± 3.61	0.16 ± 0.01	9.48 ± 3.36
	6	2.17	7.43	0.1	5.34

a The total NO release from the sample over time.

b The highest release burst from the sample.

c The time taken for a sample to achieve [NO]max.

d The total duration of release for a sample.

*T* = 0: the [NO]tot release in LB was found to be proportional to GNP/NO concentration, with increasing concentrations leading to an increase in [NO]tot release (GNP/NO2.5 (14.55 ± 11.21 μmol mg^−1^), GNP/NO5 (19.09 ± 0.67 μmol mg^−1^) and GNP/NO7 (19.96 ± 1.76 μmol mg^−1^)), unlike the results revealed under nutrient-poor conditions.

*T* = 3: at *t* = 3, a drastic decrease in the [NO]tot and [NO]max release for all GNP/NO concentrations was observed. A decrease was also seen in the [NO]td release for GNP/NO7 (9.48 ± 3.36 h), however GNP/NO2.5 (7.50 ± 7.23 h) and GNP/NO5 (7.57 ± 5.03 h) remained largely unchanged.

*T* = 6: a further reduction in [NO]tot, [NO]max [NO]td was observed for all GNP/NO concentrations at *t* = 6 compared to *t* = 0, though [NO]tmax remained largely unchanged throughout the test period.

#### 3.6.2 NO release TSB

To further investigate the effects of biologically relevant media on the NO release kinetics of GNP/NO, the NO release from GNP/NO was measured in TSB. The NO release profiles of GNP/NO2.5, GNP/NO5 and GNP/NO7 in TSB at *t* = 0 is shown in Fig. 4, and the calculated NO release kinetics at each time point are summarised in Table 3.

**Table 3:** Stability of NO release kinetics from GNP/NO in TSB

Sample	Month	[NO]tot^a^ (μmol mg^−1^)	[NO]max^b^ (nmol mg^−1^)	[NO]tmax^c^ (min)	[NO]td^d^ (h)
GNP/NO2.5	0	12.41 ± 0.48	10.48 ± 4.48	0.19 ± 0.06	9.29 ± 0.84
	3	8.15 ± 2.96	17.40 ± 9.16	0.28 ± 0.12	9.09 ± 0.44
	6	2.80	10.78	0.19	2.01
GNP/NO5	0	11.35 ± 1.70	17.48 ± 1.18	0.41 ± 0.12	3.74 ± 1.92
	3	4.92 ± 1.18	14.51 ± 8.20	0.17 ± 0.05	6.05 ± 3.85
	6	8.53	6.94	0.21	11.82
GNP/NO7	0	37.92 ± 21.60	45.63 ± 24.94	0.20 ± 0.06	12.57 ± 4.84
	3	2.47 ± 1.67	3.81 ± 0.70	0.89 ± 0.54	4.53 ± 4.27
	6	5.71	5.18	0.23	11.80

a The total NO release from the sample over time.

b The highest release burst from the sample.

c The time taken for a sample to achieve [NO]max.

d The total duration of release for a sample.

*T* = 0: similar to the results in LB, with increasing GNP/NO concentration, the [NO]tot and [NO]max release also increased in TSB, however interestingly this was not observed for [NO]td.

*T* = 3: following 3 months of storage the [NO]tot release substantially decreased for GNP/NO2.5, GNP/NO5 and GNP/NO7, 8.15 ± 2.96 μmol mg^−1^, 4.92 ± 1.18 μmol mg^−1^ and 2.47 ± 1.67 μmol mg^−1^, respectively.

*T* = 6: at *t* = 6, the [NO]tot release for GNP/NO2.5 decreased further compared to the *t* = 0 and *t* = 3 timepoints (2.80 μmol mg^−1^), however interestingly this was not seen for the GNP/NO5 (8.53 μmol mg^−1^) or GNP/NO7 (5.71 μmol mg^−1^) samples, which were found to increase slightly compared with the *t* = 3 timepoint.

#### 3.6.3 NO release in DMEM

As investigations into the cytotoxicity of GNP/NO were undertaken in DMEM, the NO release of GNP/NO was measured in the medium to ascertain its effects on the NO release kinetics. Table 4 summarises the calculated NO release kinetics for GNP/NO2.5, GNP/NO5 and GNP/NO7 and the NO release profiles at *t* = 0 are shown in Fig. 4.

**Table 4:** Stability of NO release kinetics from GNP/NO in DMEM

Sample	[NO]tot^a^ (μmol mg^−1^)	[NO]max^b^ (nmol mg^−1^)	[NO]tmax^c^ (min)	[NO]td^d^ (h)
GNP/NO2.5	10.22 ± 3.56	6.12 ± 3.28	0.25 ± 0.08	7.98 ± 1.03
GNP/NO5	6.86 ± 3.13	15.31 ± 10.13	0.97 ± 1.45	7.93 ± 0.89
GNP/NO7	3.08 ± 0.73	5.74 ± 3.67	0.17 ± 0.06	6.44 ± 1.13

a The total NO release from the sample over time.

b The highest release burst from the sample.

c The time taken for a sample to achieve [NO]max.

d The total duration of release for a sample.

As GNP/NO demonstrated no cytotoxicity at *t* = 0, the release of NO from GNP/NO in DMEM was measured at *t* = 0, alone. Similarly to results in PBS, the [NO]tot release in DMEM was not proportional to GNP/NO concentrations, it was found that GNP/NO2.5 had a [NO]tot of 10.22 ± 3.56 μmol mg^−1^ and GNP/NO5 and GNP/NO7 released 6.86 ± 3.13 μmol mg^−1^ and 3.08 ± 0.73 μmol mg^−1^, respectively.

### 3.7 Antimicrobial efficacy GNP/NO under nutrient poor conditions

*Escherichia coli*, *Staphylococcus aureus* and *Candida albicans* are prevalent microorganisms responsible for many healthcare-associated infections.^31^ To evaluate the antimicrobial efficacy of GNP/NO, and to determine the optimal concentration which retains its effectiveness after prolonged storage, we tested three different of GNP/NO (GNP/NO2.5, GNP/NO5 and GNP/NO7). The antimicrobial efficacy of the nanoparticles under nutrient-poor conditions (PBS) were evaluated at two time points: 4 h, which corresponds to the burst release of NO, and 24 h representing the total time that the NO release was measured. After each time period, the remaining microorganisms were calculated (CFU mL^−1^) and compared to a bacterial control which consisted of bacterial suspension alone. The shelf life of GNP/NO was also evaluated by performing the antimicrobial assay at three points: *t* = 0 (initial measurement), and after *t* = 3 months, and *t* = 6 months of storage.

**Fig. 6:**
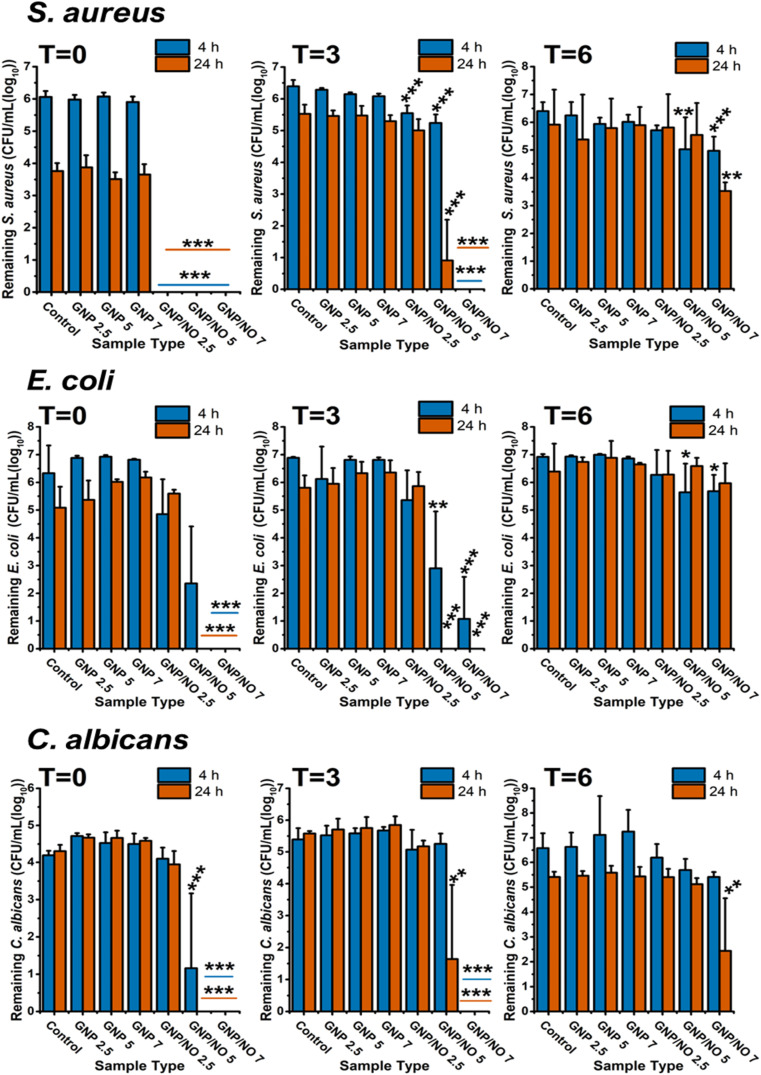
The antimicrobial efficacy of GNP/NO under nutrient-poor conditions over time. Total remaining viable microorganisms after 4 (blue) and 24 h (red) of incubation with GNP/NO2.5, GNP/NO5 or GNP/NO7 in nutrient-poor conditions (PBS). Non-functional gelatin controls (GNP) of the same weight and a positive control (control) of bacteria alone are also displayed. Statistical significance was assessed using one-way ANOVA and significance is indicated by asterisks * (*p* < 0.05), ** (*p* < 0.001) and *** (*p* < 0.0001). *n* = 3.

As shown in Fig. 6, all GNP/NO concentrations completely eradicated *S. aureus* within 4 h under nutrient-poor conditions. At *t* = 3, the antimicrobial efficacy of GNP/NO decreased, with only GNP/NO7 causing complete eradication of *S. aureus* at 4 h. After 24 h however, incubation with GNP/NO5 demonstrated a significant reduction in *S. aureus* (4.3 log). Interestingly, the antimicrobial efficacy of GNP/NO significantly decreased at *t* = 6, and GNP/NO was unable to completely eradicate *S. aureus*, although GNP/NO7 did cause a 2.36 log reduction after 24 h.

The antimicrobial efficacy of GNP/NO against *E. coli* at *t* = 0, *t* = 3 and *t* = 6 is shown in Fig. 6. Surprisingly unlike *S. aureus*, GNP/NO2.5 was revealed to be ineffective against *E. coli*, and was unable to significantly reduce the bacterium at any timepoint. However, incubation with both GNP/NO5 and GNP/NO7 caused complete eradication of *E. coli* after 24 h at both *t* = 0 and *t* = 3. A significant reduction in the antimicrobial efficacy was observed at *t* = 6, with GNP/NO5 and GNP/NO7 only reducing *E. coli* by 1.28 and 1.24 log reductions, respectively after 4 h.

In addition to examining the antibacterial properties of GNP/NO, we extended our research to examine its antifungal properties, through exposure of GNP/NO to *C. albicans*, a prevalent yeast species.

Across all time points, it was evident that a higher dosage of GNP/NO (*i.e.* ≥GNP/NO5) was required to significantly reduce *C. albicans*, as exposure to GNP/NO2.5 proved to be ineffective against the fungi (Fig. 6). At *t* = 0, both GNP/NO5 and GNP/NO7 led to the complete eradication of the fungi after 24 h. Similar to results observed against *S. aureus* and *E. coli* at *t* = 3, it was found that the antimicrobial efficacy of GNP/NO decreased against *C. albicans* over time, and only GNP/NO7 caused complete eradication of the fungi at either 4 h or 24 h. This reduction in efficacy was shown to decrease further at *t* = 6, as only the GNP/NO7 sample elicited a significant reduction in *C. albicans* (3 log) after 24 h.

### 3.8 Antimicrobial efficacy of GNP/NO in nutrient rich conditions

This study has demonstrated that media significantly influences the NO release kinetics from biomaterials, consistent with previous reports.^26^ For this reason, we also investigated how changes in NO release kinetics affected the antimicrobial efficacy of GNP/NO, by assessing its activity in two biologically relevant media, LB and TSB. The antimicrobial efficacy of GNP/NO was tested against *S. aureus* and *E. coli* in LB, and *C. albicans* in TSB after 4 h and 24 h incubation. To evaluate the longevity of GNP/NO's antimicrobial efficacy, the assays were conducted at three points: *t* = 0 (initial measurement), and after three months (*t* = 3) and after 6 months (*t* = 6) of storage.

**Fig. 7:**
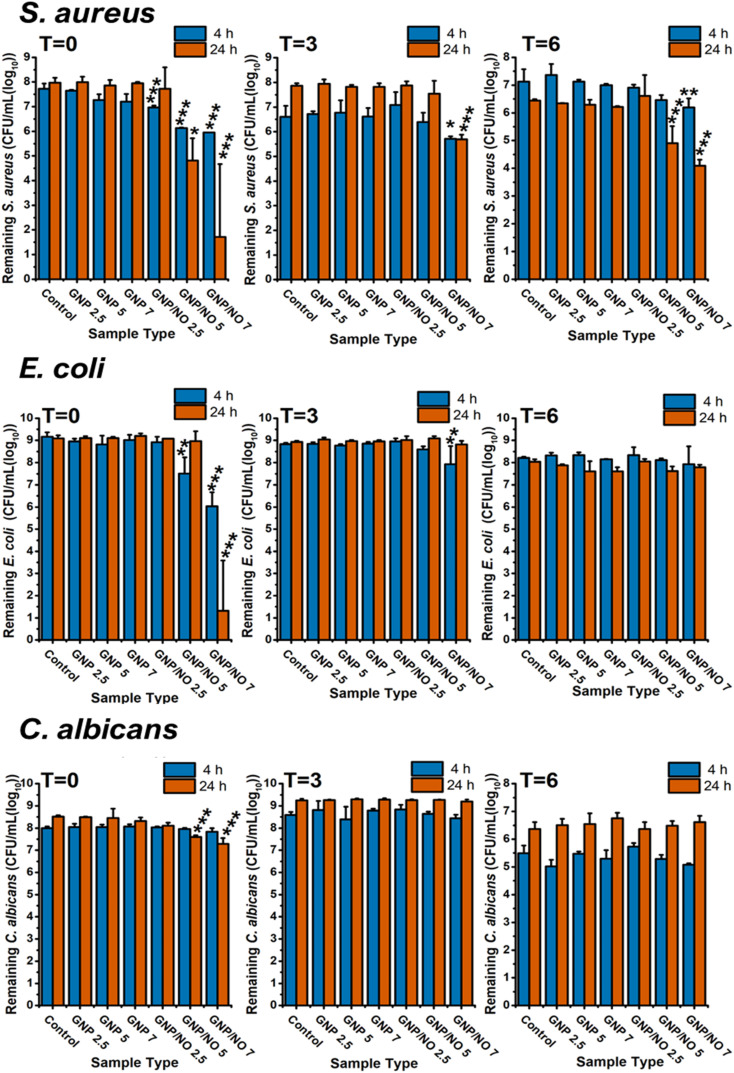
The antimicrobial efficacy of GNP/NO under nutrient-rich conditions over time. Total remaining viable microorganisms after 4 (blue) and 24 h (red) incubation with GNP/NO2.5, GNP/NO5 or GNP/NO7 in nutrient-rich conditions (LB for *S. aureus* and *E. coli* and TSB for *C. albicans*). Non-functionalised gelatin controls (GNP) of the same weight and a positive control (control) of bacteria alone are also displayed. Statistical significance was assessed using one-way ANOVA and significance is indicated by asterisks * (*p* < 0.05), ** (*p* < 0.001) and *** (*p* < 0.0001). *N* = 3.

At *t* = 0 (Fig. 7), 4 h incubation with GNP/NO2.5 (0.8 log), GNP/NO5 (1.6 log) and GNP/NO7 (1.8 log) all demonstrated significant antimicrobial efficacy against *S. aureus* under nutrient-rich conditions (LB). Interestingly however, a regrowth in *S. aureus* was observed for GNP/NO2.5 after 24 h. Similar to results observed under nutrient-poor conditions the antimicrobial efficacy of GNP/NO was found to decrease over time, with only GNP/NO7 demonstrating a 0.9 log reduction at 4 h and a 2.17 log reduction after 24 h under nutrient-rich conditions at *t* = 3. Interestingly, at *t* = 6 both GNP/NO5 (1.5 log) and GNP/NO7 (2.4 log) led to a reduction in *S. aureus* after 24 h of incubation.

It was revealed that under nutrient-rich conditions (LB), both GNP/NO5 (1.7 log) and GNP/NO7 (3.1 log) significantly reduced *E. coli* at 4 h (Fig. 7), with a further reduction of 7.8 log reduction by GNP/NO7 after 24 h. A significant reduction to the antimicrobial efficacy of GNP/NO was observed at both *t* = 3, where GNP/NO7 resulted in a 0.9 log reduction after 4 h, and at *t* = 6 GNP/NO was no longer effective against *E. coli*.

Investigation of the antimicrobial efficacy of GNP/NO against *C. albicans* under nutrient-rich conditions (TSB) revealed that at *t* = 0, both GNP/NO5 (0.9 log) and GNP/NO7 (1.2 log) caused a significant reduction in the CFU mL^−1^ counts of *C. albicans* after 24 h. However, no significant antimicrobial activity was exhibited by GNP/NO after this time point.

### 3.9 Antibiofilm efficacy of GNP/NO under nutrient-poor conditions

Approximately 80% of healthcare-associated infections are the result of biofilms rather than a planktonic infection. Moreover, the increased resistance of biofilms to antibiotics can lead to prolonged treatments, poor patient outcomes, and high reinfection rates.^32^ Therefore, considering its clinical relevance the antibiofilm capability of GNP/NO was evaluated against established *E. coli* and *S. aureus* biofilms. The antibiofilm efficacy of GNP/NO against *C. albicans* was not assessed due to the strains' inability to form biofilms.

Biofilms were established over a 24 h period and subsequently washed to remove planktonic organisms. The established biofilms were then incubated in nutrient-poor conditions (PBS) with GNP/NO, and the remaining viable microorganisms were counted after 4 and 24 h of incubation. The antibiofilm efficacy of the particles was investigated after 0, 3 and 6 months of storage to evaluate their shelf life.

**Fig. 8:**
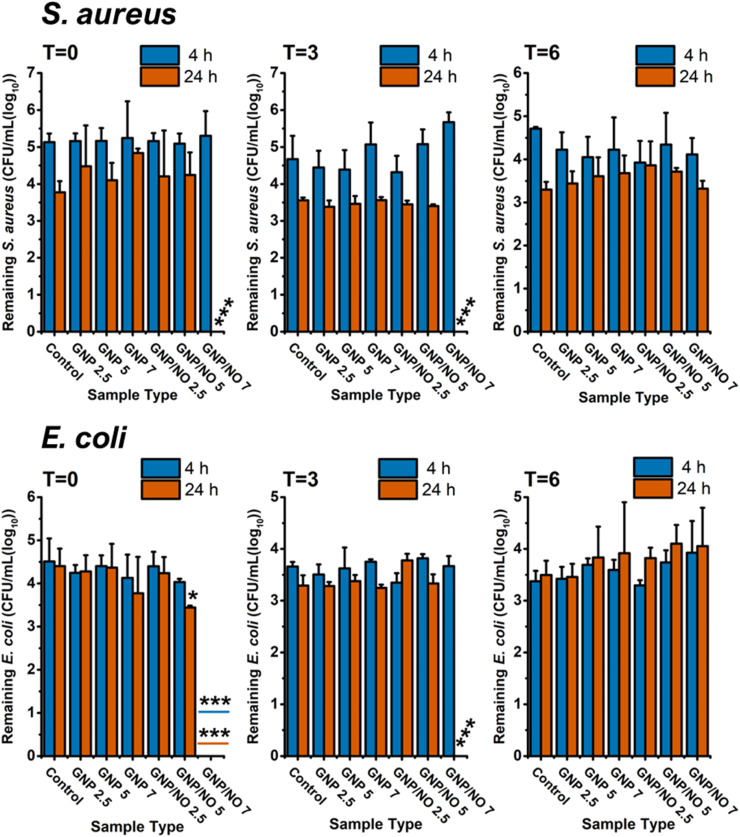
Antibiofilm efficacy of GNP/NO under nutrient-poor conditions. Total remaining viable microorganisms after 4 (blue) and 24 h (red) of incubation with GNP/NO2.5, GNP/NO5 or GNP/NO7 in PBS. Non-functionalised gelatin controls (GNP) of the same weights and a positive control (control) of bacteria alone, are also displayed. Antibiofilm efficacy of GNP/NO against *S. aureus* and *E. coli* was conducted after 0, 3 and 6 months of storage indicated by *T* = 0, *T* = 3 and *T* = 6. Statistical significance was assessed by one-way ANOVA and significance is indicated by asterisks * (*p* < 0.05), ** (*p* < 0.001) and *** (*p* < 0.0001).

The results showed that only 24 h incubation with the highest concentration, GNP/NO7 led to significant dispersal of *S. aureus* biofilms at *t* = 0 and *t* = 3 under nutrient-poor conditions (Fig. 8), suggesting a higher NO dosage may be required against *S. aureus* biofilms compared to planktonic *S. aureus*. At *t* = 6, GNP/NO particles no longer exhibited antibiofilm activity against established *S. aureus* biofilms.

Fig. 8 illustrates the antibiofilm efficacy of GNP/NO against the established *E. coli* biofilms under nutrient-poor conditions. At *t* = 0, GNP/NO7 caused complete dispersion of *E. coli* biofilms after 4 h of incubation, and incubation with GNP/NO5 led to a 0.9 log reduction in *E. coli* biofilms after 24 h. At *t* = 3, only GNP/NO7 resulted in the complete dispersion of *E. coli* biofilms after 24 h, however at *t* = 6 the nanoparticles were no longer effective against established *E. coli* biofilms, similar to results seen against *S. aureus* biofilms.

### 3.10 Antibiofilm efficacy of GNP/NO under nutrient-rich conditions

In addition to investigating the antibiofilm efficacy of GNP/NO under nutrient-poor conditions, the antibiofilm efficacy of GNP/NO was assessed under nutrient-rich conditions. The antibiofilm capabilities of GNP/NO was evaluated against established *S. aureus* or *E. coli* biofilms in LB, after either a 4 h or 24 h incubation period. To further assess the shelf-life of GNP/NO, the experiments were repeated after 3 and 6 months of storage.

**Fig. 9:**
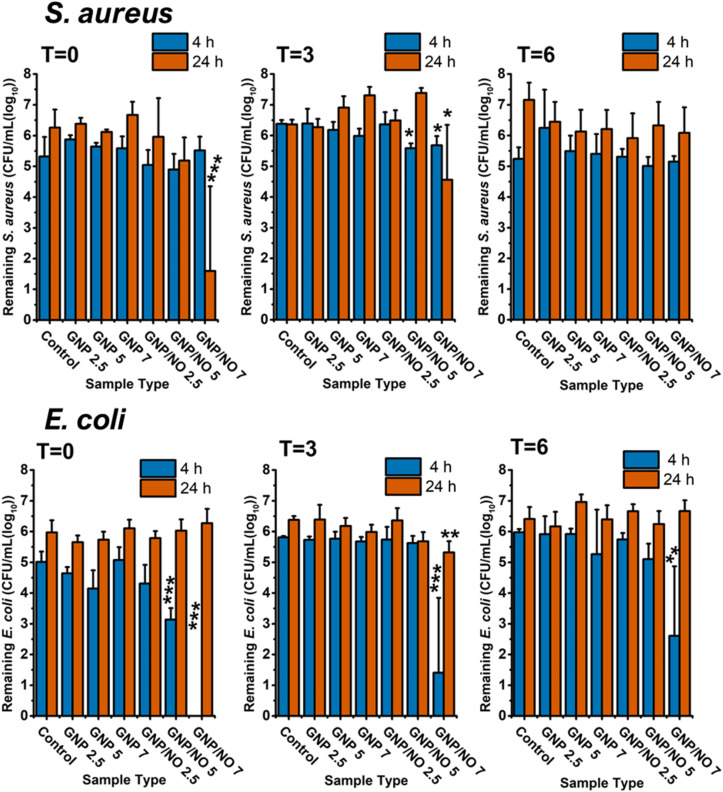
Stability of antibiofilm efficacy of GNP/NO under nutrient-rich conditions. Total remaining viable microorganisms after 4 (blue) and 24 h (red) incubation with GNP/NO2.5, GNP/NO5 or GNP/NO7 in PBS. Non-functionalised gelatin controls (GNP) of the same weights and a positive control (control) of bacteria alone, are also displayed. Antibiofilm efficacy of GNP/NO against *S. aureus* and *E. coli* was conducted after 0, 3 and 6 months of storage, indicated by *T* = 0, *T* = 3 and *T* = 6. Statistical significance was assessed by one-way ANOVA and significance is indicated by asterisks * (*p* < 0.05), ** (*p* < 0.001) and *** (*p* < 0.0001).

Fig. 9 shows the effects of GNP/NO on established *S. aureus* biofilms under nutrient-rich conditions. GNP/NO7 caused significant biofilm disruption after 24 h, reducing the biofilm by 4.7 log. At *t* = 3, both GNP/NO5 and GNP/NO7 reduced *S. aureus* biofilms within 4 h, resulting in 0.8 log and 0.7 log reductions, respectively. However, biofilms incubated with GNP/NO5 reformed after 24 h. At *t* = 6, GNP/NO did not demonstrate antibiofilm activity against established *S. aureus* biofilms.

The antibiofilm capabilities of GNP/NO against established *E. coli* biofilms under nutrient-rich conditions, over a 6-month period were also investigated (Fig. 9). It was revealed that both GNP/NO5 (1.9 log) and GNP/NO7 (complete dispersal) significantly reduced the presence of *E.coli* biofilms at 4 h, however, a biofilm reformation was observed in both samples at 24 h. The results at *t* = 3, showed that incubation with GNP/NO7 caused a 4.4 log biofilm reduction after 4 h. Interestingly, despite reformation of the biofilm at 24 h, GNP/NO7 still remained significantly lower than the control (1.1 log). Surprisingly, after 6-months of storage, GNP/NO7 maintained its antimicrobial efficacy leading to a 3.4 log reduction to the *E.coli* biofilms after 4 h, although the biofilm fully reformed after 24 h.

### 3.11 Cytotoxicity assay

To assess the cytocompatibility of GNP/NO as a potential therapeutic agent, we evaluated its effect on the viability of murine fibroblasts, using the L929 cell line.

**Fig. 10:**
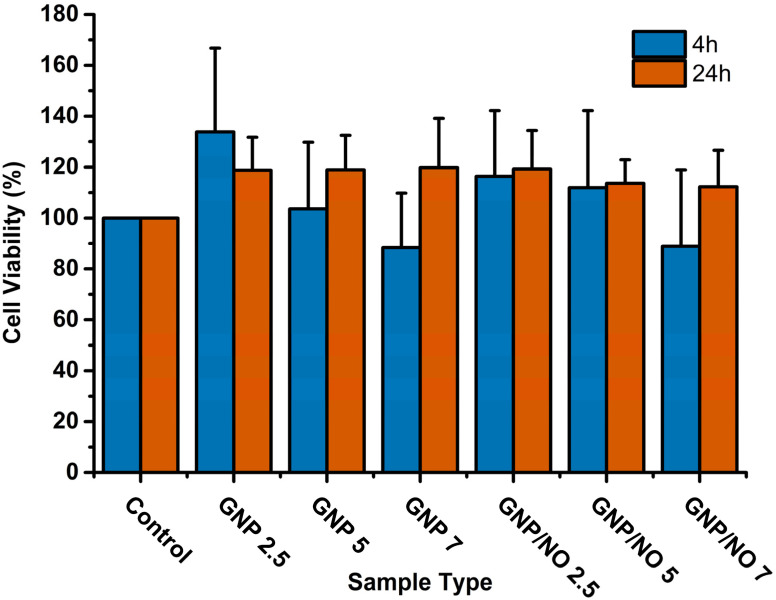
Cytotoxicity of GNP/NO. The cell viability (%) of L929 cells after incubation with GNP/NO leachates formed after 4 h (blue) or 24 h (red) incubation in DMEM. The positive control displays the results from the incubation of the cells alone. The absorbance at 570 nm was used to measure the light intensity.

This study assessed the effects of GNP/NO leachates on L929 cells formed by the incubation of GNP/NO in DMEM for 4 and 24 h (Fig. 10). As no sample reduced the cell viability below 70%, it suggests that GNP/NO is biocompatible based on the ISO10993-22 protocol. Surprisingly, many samples demonstrated an increase in cell viability, as much as 35% for the GNP2.5, 4 h leachate.

## 4. Discussion

The increasing rise of antimicrobial resistance has created an urgent need for novel antimicrobials. Nitric oxide is a promising broad-spectrum antimicrobial with multimechanistic biocidal properties, and therefore may offer a potential solution to this problem. In this study, we synthesised gelatin nanoparticles tethered to an NO donor, *N*-diazeniumdiolates (GNP/NO), and assessed their feasibility as a novel antimicrobial treatment. We have evaluated the payload and release kinetics of NO from GNP/NO in various biologically relevant media, to ascertain its impact on the antimicrobial and antibiofilm efficacy of the nanoparticles. We investigated this further by assessing the NO payload, release, and antimicrobial and antibiofilm efficacies against three important healthcare associated microorganisms, to fully elucidate the stability and longevity of the nanoparticles. Finally, the cytotoxicity of the particles was analysed to ascertain their potential as a novel antimicrobial therapeutic.

The synthesis of the nanoparticles was carried out using a two-step desolvation method, which resulted in smooth, homogeneous particles with a diameter of ∼200 nm and a PDI of 0.09. The PDI indicates the size distribution within a population, with a PDI ≥1.00 representing a polydisperse particle size distribution, and a PDI of 0.00 indicating a monodispersed size population.^33^ Within polymer nanomaterial research a PDI ≤0.2 is generally considered acceptable for use in medical therapeutics,^34^ suggesting that the GNP synthesised in this study form relatively uniform particle size distributions (monodispersed) within the therapeutic range. Additionally, comparison of different synthesis batches found minimal variation in the size distribution (Fig. S1†), indicating the 2-step desolvation method to be a robust and repeatable method for GNP synthesis.

Interestingly, a discrepancy was identified between particle diameter measurements obtained through DLS (226.9 ± 18.8 nm) compared to SEM (190 ± 8 nm), approximately 36.3 nm difference in diameter. This coincides with previous reports by Hassani Besheli^35^ which showed the hydrodynamic diameter of GNP, measured by DLS to be larger than SEM measurements. This is likely due to the hydrophilic nature of gelatin,^36^ which cause the particles to swell in aqueous solutions. To further validate this, lyophilised GNP were rehydrated and the hydrodynamic diameter was remeasured every 5 min over a 24 h period *via* DLS (Fig. S2†), the results revealed the GNP diameter to increase by ∼40 nm within the first 5 min of measurements, closely aligning with the variations observed between DLS and SEM measurements.

The pH of all media in this study was tested to evaluate their effects on the NO release from GNP/NO. This is essential as NO release is dependent on pH, and the different buffering capacities of each medium can significantly affect the decomposition of *N*-diazeniumdiolates. All media examined in this study exhibited a pH between 6.9–7.5, however, the addition of GNP/NO (GNP/NO7), led to a decrease in pH. This is due to the formation of reactive oxygen species (ROS) and reactive nitrogen species (RNS), which are rapidly produced by the reaction of NO in the presence of O_2_ and H_2_O. One product of this reaction is N_2_O_3_, this can react with H_2_O to form H^+^ and NO_2_^−^, increasing the concentration of H^+^ present, thus lowering the pH of the medium.^37^ Subsequent pH measurements at 2, 4, 18, and 24 h revealed that the pH of each medium gradually returned to its original pH, which was likely due to the presence of buffers in the media.

Chemiluminescence is widely regarded as the gold standard for NO detection due to its sensitivity with a detection range of 0.5 ppb to 500 ppm,^38^ and a detection rate of 10^−7^ L mol^−1^ s^−1^, allowing for near real-time analysis NO analysis. Given these advantages, this method was chosen to measure the NO payload and release kinetics of GNP/NO (GNP/NO2.5, GNP/NO5 and GNP/NO7) under nutrient-poor and nutrient-rich conditions. The choice of medium significantly influenced the NO release with considerable differences in both the total release ([NO]tot) and initial burst release ([NO]max) concentrations. Interestingly, at *t* = 0 the [NO]tot release from GNP/NO2.5, was as follows: PBS < DMEM < TSB < LB, with PBS releasing the least. This strongly correlates with the initial pH measurements of each medium, where PBS showed the highest pH (pH 7.83 ± 0.07), followed by DMEM (pH 7.42 ± 0.02), TSB (pH 7.12 ± 0.16), and LB (pH 6.93 ± 0.19). These findings align with previous studies Salmon^39^ which indicated that NO release from *N*-diazeniumdiolates is mediated by pH. Furthermore, Tai *et al.*^40^ demonstrated that even small decreases in pH (from 7 to 6) can increase NO release by 2-3-fold. In this case, a higher pH would indicate a lower proton concentration, leading to lower protonation of *N*-diazeniumdiolates, resulting in less NO being released and a reduced [NO]tot.

The shelf life of GNP/NO was determined by assessing the NO payload from the particles in PBS, LB, and TSB at *t* = 0, *t* = 3 and *t* = 6 to evaluate its practicality as a therapeutic agent. The NO release kinetics in DMEM were not evaluated over time, as GNP/NO was found to be cytocompatible at *t* = 0, and therefore it was deemed unnecessary to assess the NO release past this point. A general decrease in the [NO]tot concentrations was observed over time for all media tested, with an average reduction of 75% between *t* = 0 and *t* = 6. Though, the decomposition of *N*-diazeniumdiolate is predominantly pH-mediated, it can also occur through thermal dissociation,^39^ therefore throughout this study GNP/NO was stored at −20 °C to improve the storage capabilities of the nanoparticles. Evidence suggests that the stability of *N*-diazeniumdiolates can be prolonged by storage in a nitrogen environment. Batchelor^41^ demonstrated that lipophilic dialkyldiamine-based diazeniumdiolates maintained 99% of their NO release capability when stored in a dry nitrogen environment, compared to 62% when stored under ambient conditions after 4 weeks. The authors attributed the slow decomposition to the presence of water vapour within the polymer, resulting in NO release from the biomaterial, which may explain the decline in [NO]tot concentrations over time. Despite this decrease, the results of this study have shown GNP/NO continues to release NO for up to six-months when stored at −20 °C. Further research is required to fully understand the effects of storage conditions on GNP/NO, and to improve the nanoparticle's shelf life.

To evaluate the broad-spectrum antimicrobial potential of GNP/NO, the antimicrobial efficacy of GNP/NO2.5, GNP/NO5 and GNP/NO7 was assessed against three common healthcare-associated pathogens, *S. aureus*, *E. coli* and *C. albicans*. These concentrations were evaluated over a six-month to determine the longevity of their effectiveness. Additionally, as our results indicated that the medium significantly impacted the NO release kinetics from GNP/NO, we also examined the antimicrobial efficacy of the nanoparticles under both nutrient-poor and nutrient rich conditions to determine the effect on the nanoparticles' performance.

Although, the NO release kinetics showed the [NO]tmax of GNP/NO to be <1 min, an incubation period of up to 24 h was often necessary for the antimicrobial GNP/NO to take effect. This discrepancy is expected, as NO is a free radical with a short half-life,^42^ therefore the molecule itself does not typically act on the microorganism. Instead, its antimicrobial activity primarily arises from the formation of RNS and ROS through interactions with oxygen and superoxide (˙O_2_^−^). This effect is particularly evident when comparing the antimicrobial efficacy of GNP/NO at 4 and 24 h. The initial reduction observed at 4 h results from the oxidative and nitrosative stress within the cell, driven by ROS and RNS such as peroxynitrite (ONOO^−^), nitrogen dioxide (NO_2_) and dinitrogen tetroxide (N_2_O_3_). The further decrease at 24 h is largely attributed to the secondary effects of these reactive species, including DNA damage, inactivation of proteins and lipid peroxidation. These processes ultimately lead to cell death, even after NO concentrations have diminished.^43^

Nitric oxide exhibits antimicrobial and antibiofilm properties in a concentration dependent manner, concentrations of >1 μM are generally considered sufficient to exert bactericidal effects. Conversely, sublethal NO concentrations of ∼0.5 nM NO induce the dispersal of biofilms, causing bacteria to revert to their planktonic state.^26^ In our study, the NO release kinetics observed suggest GNP/NO can achieve concentrations within these effective ranges, indicating both antibiofilm and antimicrobial capabilities. However, the antimicrobial results observed are more complex, suggesting additional factors may influence the antimicrobial and antibiofilm efficacies of GNP/NO.

Initial results at *t* = 0 revealed there to be differences in the antimicrobial efficacy of GNP/NO between microorganisms, it was found that the nanoparticles were more effective against *S. aureus* compared to *E.coli* or *C. albicans*, as the lowest concentration (GNP/NO2.5) was able to significantly reduce the bacteria at *t* = 0, under both nutrient-poor and nutrient-rich conditions. This coincides with reports in the literature, showing NO to be more effective against Gram positive bacteria.^44,45^ This can be attributed to the ability of Gram negative bacteria such as *E. coli* to synthesise flavohemoglobins, which convert NO radicals to NO_3_^−^ ions *via* nitrosylation and can counteract the nitrosative stress caused by RNS produced by NO.^46^ Although, some studies have reported the contrary, suggesting Gram negative bacteria are more susceptible to NO,^47–49^ it is likely that the susceptibility of bacteria to ROS and RNS is independent of a bacterium's Gram classification and is affected more by the species itself, the NO dose concentration, and the method of NO delivery.

The antimicrobial efficacy of GNP/NO was evaluated against *C. albicans*, a common healthcare-associated pathogen known to cause candidiasis and bloodstream infections.^50^ The study found that *C. albicans* exhibited a greater resistance to GNP/NO under nutrient-poor conditions compared to *S. aureus* and *E. coli*, as higher concentrations of GNP/NO (>GNP/NO5) were required to significantly reduce the fungi, consistent with previous studies.^51^ The antifungal mechanisms of NO have been found to be similar to its antibacterial actions, with the ROS and RNS generated by NO interacting with DNA, lipid membranes and proteins^9,52^ ultimately leading to cell death. As the lipid cell membrane in yeast are encased within a cell wall,^53^ this may provide some protection from cell damage through lipid peroxidation by increasing the distance that ROS and RNS, generated by NO must travel to reach the membrane.

Given the variations in NO payload observed by GNP/NO in nutrient-rich conditions, we extended our investigation to assess the antimicrobial efficacy of GNP/NO using two biologically relevant media, LB and TSB. The shelf life of the particles was again investigated over a six-month period (*t* = 0, *t* = 3 and *t* = 6).

Although GNP/NO released higher [NO]tot concentrations under nutrient-rich conditions (LB and TSB) compared to nutrient-poor conditions (PBS), suggesting a potential increase to its antimicrobial capabilities, the results did not reflect this. In contrast, GNP/NO exhibited reduced antimicrobial activity under nutrient-rich conditions. While incubation with GNP/NO under nutrient-rich led to significant microbial reductions, the particles did not achieve complete eradication.

This reduced antimicrobial efficacy in nutrient-rich conditions may be due to the presence of proteins, trace metal ions and amino acids present, which have been shown to inhibit or sequester NO. For instance, LB contains tryptone, which comprises many amino acids, including tryptophan, cysteine and methionine, all of which have been identified as free-radical scavengers or antioxidants.^54^ Furthermore, TSB contains high concentrations of glucose, a known NO scavenger across both the animal and plant kingdom, which plays a role in modulating signalling pathways.^55^ Therefore, it can be hypothesised that these scavengers in LB and TSB quench the NO radicals, along with any generated RNS or ROS before they induce nitrosative and oxidative stress in the organisms.

Furthermore, studies have indicated that nutrient-poor conditions can increase a microorganism's susceptibility to antimicrobial agents.^56^ This increased susceptibility to GNP/NO under nutrient-poor conditions may occur due to a lack of resources. For example, superoxide dismutase (SOD), an enzyme that mitigates oxidative stress in microorganisms, requires Mn, Fe, Cu or Zn ions for its catalytic activity.^57^ However, these ions are also essential for protein synthesis and lipid metabolism in microorganisms.^58^ Due to resource limitations in nutrient-poor conditions, ions that would typically be used to help counteract nitrosative and oxidative stress are instead preferentially used to maintain minimum or essential metabolic functions,^59^ making microorganisms more susceptible to GNP/NO under nutrient-poor conditions. These results highlight the importance of assessing the antimicrobial efficacy of NO-releasing biomaterials in various environments to fully understand their potential.

This study also assessed the antimicrobial efficacy of GNP/NO after 0, 3 and 6 months of storage. The antimicrobial efficacy of GNP/NO decreased over time in both nutrient-poor and nutrient-rich conditions against all microorganisms tested, consistent the NO release kinetics results. Despite this reduction, GNP/NO7 was still found to significantly reduce planktonic *S. aureus* under nutrient-poor conditions at *t* = 6, indicating that GNP/NO still maintained partial antimicrobial efficacy after 6 months of storage.

Given that many hospital-associated chronic infections are often the result of biofilm formation, we investigated the antibiofilm efficacy of GNP/NO. This study assessed the ability of GNP/NO particles to disperse established *S. aureus* or *E. coli* biofilms, under both nutrient-poor and nutrient-rich conditions, at *t* = 0, *t* = 3 and *t* = 6.

Incubation of GNP/NO with established *S. aureus* and *E. coli* biofilms resulted in complete biofilm dispersion after 24 h under nutrient-poor conditions, up to *t* = 3. This finding is consistent with many studies demonstrating that NO is an effective biofilm dispersant.^60,61^ Interestingly, GNP/NO2.5 was unable to cause significant biofilm disruption at any time point for either *S. aureus* or *E. coli* biofilms, suggesting that higher concentrations of GNP/NO may be required to combat biofilms than planktonic bacteria.

Under nutrient-rich conditions, it was revealed that despite GNP/NO causing significant reductions in *E. coli* biofilms at 4 h, reformation was observed after 24 h. NO-mediated biofilm dispersal in Gram-negative bacteria, such as *E. coli* has been attributed to the activation of phosphodiesterase enzymes by NO, which hydrolyses cyclic-di-GMP. This reduction in cyclic-di GMP triggers a shift from a non-motile to a motile state, leading to biofilm dispersal.^62^ However this shift does not eradicate the bacteria, allowing biofilms to reform over time,^63^ aligning with the findings of this study. In contrast, NO-mediated dispersal of Gram-positive, such as *S. aureus* appears to occur independently of cyclic-di-GMP, though its exact mechanisms remains largely unknown.^60^ Notably, in this study the reformation of *S. aureus* biofilms was only observed once at *t* = 3, under nutrient-rich conditions, potentially supporting the idea that NO disperses *S. aureus* biofilms through an alternative pathway.

Finally, we study assessed the cytotoxic effects of GNP/NO on L929 cells according to ISO-10993. The results demonstrated that GNP/NO was cytocompatible, with no reduction in cell viability below 70%. Interestingly, several tested samples displayed increased cell viability. As gelatin is known to promote cell proliferation,^64^ and this observation is likely due to increased cell proliferation.

## 5. Conclusion

To conclude this study has shown that the 2-step desolvation method produces uniform spherical GNP with good homogeneity and excellent reproducibility across batches. The successful functionalisation of GNP with diazeniumdiolate leading to the formation of GNP/NO was confirmed through chemiluminescence measurements. The functionalised nanoparticles retained some antimicrobial efficacy against *S. aureus*, *E. coli* and *C. albicans* in nutrient-poor conditions even after 6 months. However, GNP/NO exhibited reduced effectiveness under nutrient-rich conditions, due to the quenching of NO radicals by tryptone and glucose present in LB and TSB. Interestingly, these results were not reflected in the NO release kinetics observed in the same media, suggesting that further investigation is necessary to ascertain the underlying mechanisms. Finally, cytotoxicity assays indicated that GNP/NO are not cytotoxic to murine fibroblasts. Additional research is warranted to assess potential applications of GNP/NO in combating antimicrobial resistance.

## Data availability

Data is available within the article or its ESI.† The data that support the findings is also available on request from the first author (Erin Myles E.Myles2@liverpool.ac.uk) and corresponding author (Dr Jenny Aveyard zippy78@liverpool.ac.uk)

## Conflicts of interest

There are no conflicts of interest to declare.

## Supplementary Material

NA-OLF-D4NA01042F-s001
