# Supplementary material for: Antimicrobial nitric oxide releasing gelatin nanoparticles to combat drug resistant bacterial and fungal infections
Source: Nanoscale Adv. 2025 Apr 8;7(10):3096–113. doi: 10.1039/d4na01042f (PMC11976662; doi:10.1039/d4na01042f)
Supplement: NA-007-D4NA01042F-s001 [file NA-007-D4NA01042F-s001.pdf]

Supplementary Information (SI) for Nanoscale Advances.
This journal is © The Royal Society of Chemistry 2025

Supporting Information

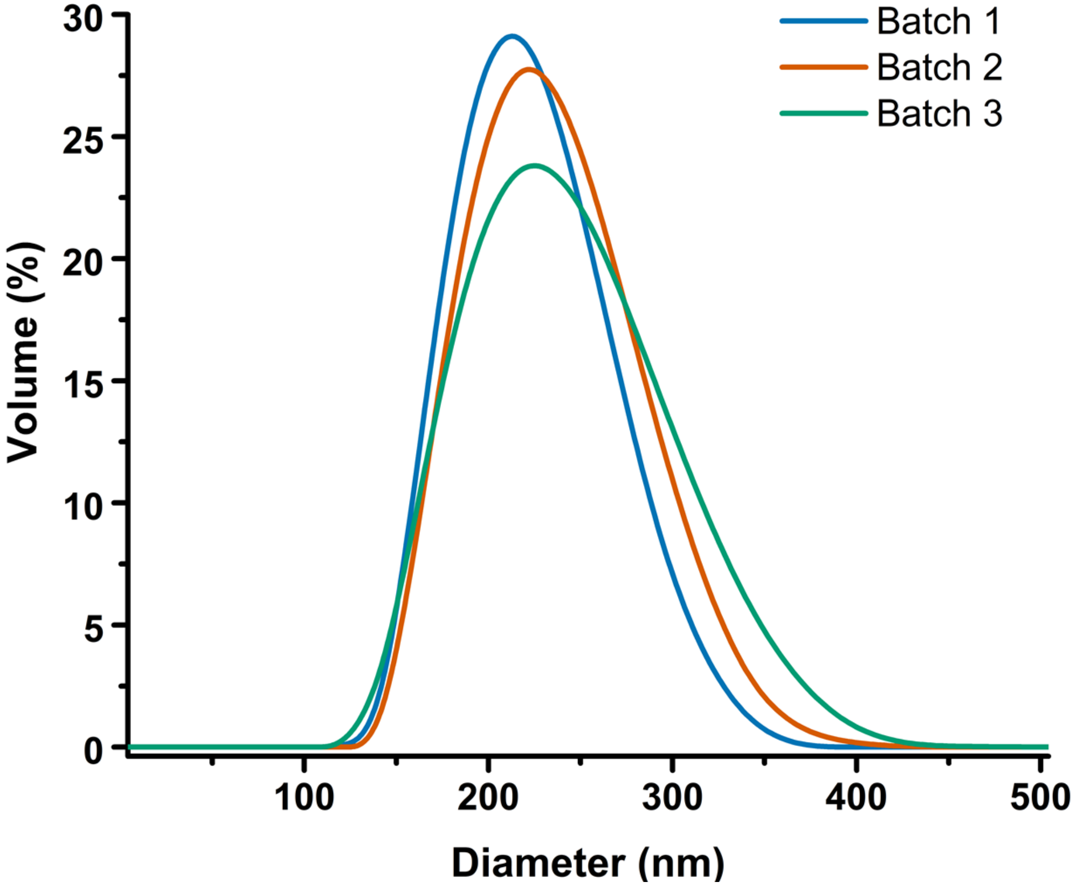

Supporting Figure 1. Interbatch variability of three batches of GNP synthesised under the same parameters. The total volume of particles (volume %) within various size distributions for three batches of GNP synthesised under normal conditions. Batch 1 (blue), Batch 2 (red) and Batch 3 (green).

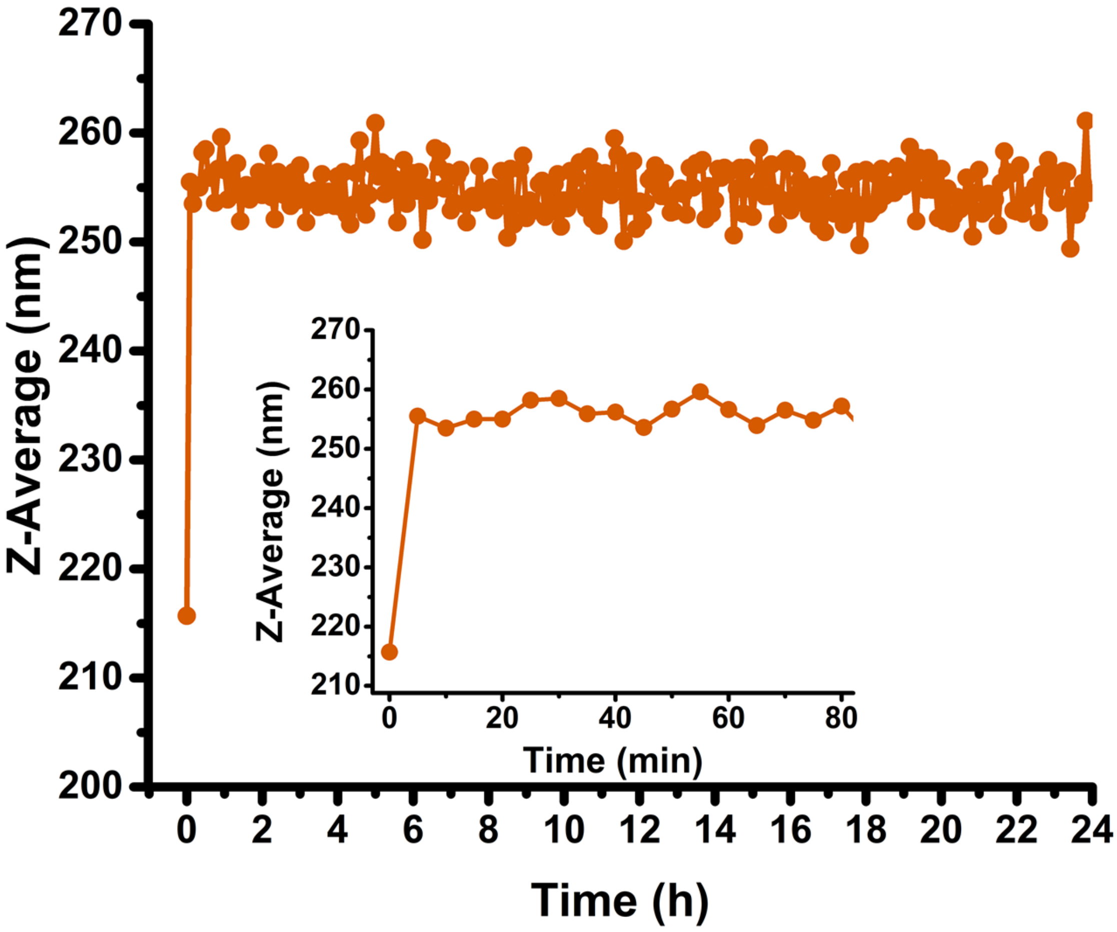

Supporting Figure 2. Swelling analysis of GNP over time measured by DLS (Z-average). The effects of water on particles diameter are shown for both pre (blue) and post (red) lyophilised particles.
